# Implicit versus explicit Bayesian priors for epistemic uncertainty estimation in clinical decision support

**DOI:** 10.1371/journal.pdig.0000801

**Published:** 2025-07-29

**Authors:** Malte Blattmann, Adrian Lindenmeyer, Stefan Franke, Thomas Neumuth, Daniel Schneider

**Affiliations:** Innovation Center Computer Assisted Surgery (ICCAS), Leipzig University, Semmelweisstraße 14, Leipzig, Germany; University of Oxford, UNITED KINGDOM OF GREAT BRITAIN AND NORTHERN IRELAND

## Abstract

Deep learning models offer transformative potential for personalized medicine by providing automated, data-driven support for complex clinical decision-making. However, their reliability degrades on out-of-distribution inputs, and traditional point-estimate predictors can give overconfident outputs even in regions where the model has little evidence. This shortcoming highlights the need for decision-support systems that quantify and communicate per-query epistemic (knowledge) uncertainty. Approximate Bayesian deep learning methods address this need by introducing principled uncertainty estimates over the model’s function. In this work, we compare three such methods on the task of predicting prostate cancer–specific mortality for treatment planning, using data from the PLCO cancer screening trial. All approaches achieve strong discriminative performance (AUROC = 0.86) and produce well-calibrated probabilities in-distribution, yet they differ markedly in the fidelity of their epistemic uncertainty estimates. We show that implicit functional-prior methods-specifically neural network ensembles and factorized weight prior variational Bayesian neural networks—exhibit reduced fidelity when approximating the posterior distribution and yield systematically biased estimates of epistemic uncertainty. By contrast, models employing explicitly defined, distance-aware priors—such as spectral-normalized neural Gaussian processes (SNGP)—provide more accurate posterior approximations and more reliable uncertainty quantification. These properties make explicitly distance-aware architectures particularly promising for building trustworthy clinical decision-support tools.

## Author summary

In this study, we address a critical challenge in applying AI to personalized medicine: models often make confident predictions even when faced with patient data unlike anything they’ve seen before. We evaluated three strategies for helping these models recognize and signal their own uncertainty, using real-world prostate cancer screening data. While all approaches performed well on familiar cases, they differed in how reliably they indicated doubt on unfamiliar patients. We discovered that methods explicitly designed to gauge how “far” a new patient’s data lies from prior examples produced far more trustworthy uncertainty estimates than techniques relying on hidden assumptions. By clearly identifying when the model is unsure, these approaches can help clinicians avoid over-reliance on AI recommendations. Our findings suggest that uncertainty-aware models could serve as safer, more transparent partners in treatment planning. Ultimately, this work takes us a step closer to AI systems that not only predict health outcomes but also responsibly signal when they might be guessing—an essential feature for trustworthy clinical decision support.

## 1. Introduction

With the adoption of electronic health records in more and more healthcare systems, huge amounts of complex heterogeneous data is provided to medical practitioners and calls to be harnessed for more personalized therapeutic approaches [1–3]. While manual examination of these large data volumes is unfeasible, data-driven techniques from the fields of statistics and machine learning (ML) are promising for exploitation through data mining, summarization, and inference. Data-driven inference may enable or aid precision medicine in a multitude of applications in medical diagnosis, disease monitoring, and outcome prognosis [4–7]. One notable application of interest is represented by personalized cancer therapy based on highly heterogeneous data including clinical diagnostic, anamnestic, and multi-omics features [8–10]. Here, data-driven approaches may assist in stratifying patients by risk and inform clinical decisions, such as in interdisciplinary tumor boards or patient participation. Prostate cancer (PCa) treatment exemplifies complex oncological decision making. Because PCa therapies can cause severe, quality-of-life–impacting side effects while many patients exhibit relatively high survival rates even without aggressive intervention, clinicians must perform personalized risk assessments that balance multiple factors [11–13]. These factors include disease severity, comorbidities and medical history, lifestyle and life circumstances, and patient preferences. Consequently, personalized PCa therapy may serve as a model for healthcare applications in which data-driven clinical decision-support (CDS) tools can improve therapeutic outcomes and optimize resource allocation. Efficient and safe adoption of ML for such decision support tasks hinges on trust—that is, on model predictions being both reliable and evidence-based [14,15]. Yet ML models frequently produce overconfident predictions when faced with inputs outside their training distribution, creating serious risks in safety-critical settings [16,17]. Regulatory frameworks for medical devices and AI-specific guidelines require manufacturers to demonstrate overall calibration, discrimination, and clinical validity on large, representative cohorts (e.g., [18–20]). However, these population-level metrics, while necessary, say nothing about the reliability of individual predictions. As patient records grow more complex and clinicians remain unaware of every nuance in a model’s training data, it becomes impractical to manually identify out-of-distribution cases. This forces practitioners into a dilemma between distrusting the model’s outputs altogether—thereby undermining CDS efficiency—or placing unwarranted trust in every prediction, risking patient safety. To resolve this, we need per-query quantification and communication of epistemic uncertainty reflecting the model’s knowledge limits-complementary to aleatoric uncertainty arising from data noise [21,22]. Depending on these measures, medical professionals may assess on a case-by-case basis to what extent to include the model predictions into their decisions. While deterministic (single-function) ML models lack awareness of epistemic uncertainty and can thus make claims without the necessary evidence [14,15], many methods for estimating epistemic uncertainty and detecting out-of-distribution (OoD) inputs have been developed to mitigate this problem. The fidelity of epistemic uncertainty estimates and OOD-detection scores depends on factors such as dataset representativeness, model architecture and capacity, the accuracy of posterior approximations or prior specifications, training procedures (e.g., regularization and optimization), and hyperparameter choices [23].

### Related work.

This paragraph focuses on related work in epistemic uncertainty estimation for medical applications. For a general overview of methods for epistemic uncertainty quantification, see Sect 2.4. Despite its potential to enhance the trustworthiness of AI-based clinical prognosis and decision support, epistemic uncertainty estimation for deep learning remains largely underexplored in medical research, with no consensus on the most suitable methods for its quantification [24]. Predominantly used methods in medical uncertainty estimation studies include Monte Carlo dropout, Bayesian neural networks, model ensembles, and variational autoencoders [25–39]. Although these methods improve upon deterministic models that entirely lack epistemic uncertainty awareness, they are often unreliable and tend to underestimate epistemic ambiguity [40–43] (see Sect 2.4). In [44], OOD regions are detected by using a classifier’s posterior probability of a “no-match” class as an instance-wise OOD score—however, this relies on a deterministic mapping prone to overconfidence. Other studies focus solely on aleatoric or total uncertainty without distinguishing uncertainty types [45–47], or estimate epistemic uncertainty only at the population level rather than instance-wise [48–50], despite the fact that these distinctions are critical for appropriate mitigation strategies. Most of the existing work targets image-based applications, with limited attention given to clinical prognosis or decision support using longitudinal patient records [24]. Despite their potential, approaches that quantify epistemic uncertainty via variational inference with explicit functional priors have seen limited exploration in medical research, with a few notable exceptions: For example, [51] propose a hybrid architecture that inputs Inception-V3–extracted features into a Gaussian process to predict diabetic retinopathy severity. Li *et al*. propose Deep Bayesian Gaussian Processes, integrating Bayesian feature-extractor networks with Gaussian processes to quantify epistemic uncertainty predicting first-onset heart failure, diabetes, and depression from electronic health records [52]. Wu *et al*. integrate a convolutional backbone with a sparse Gaussian process head for tasks such as bone age prediction and lesion localization [53]. Lindenmeyer *et al*. compare neural network ensembles and spectral-normalized neural Gaussian processes (SNGP) for epistemic uncertainty estimation in delirium risk prediction and in-hospital mortality prediction, showing that SNGP offers improved OOD detection while preserving comparable predictive accuracy [54,55]. Building on these works, our study aims to help move the medical field from prevalent but flawed techniques for epistemic uncertainty quantification toward more principled approaches based on explicit functional priors, demonstrated through a clinical prognosis use case.

### Outline.

With this study, we evaluate how well different approximate Bayesian deep learning methods quantify epistemic uncertainty in a clinical decision-support setting. Considering prostate cancer (PCa) mortality prediction on data from the Prostate, Lung, Colorectal, and Ovarian (PLCO) Cancer Screening Trial [56,57], we compare techniques that rely on implicit functional priors—specifically stochastic weight Bayesian neural networks (BNN) and neural networks ensembles (ENN)—with approaches that explicitly enforce functional prior properties, such as spectral-normalized neural Gaussian processes (SNGP). Ultimately, this work aims to identify and promote methods in the medical domain that deliver more reliable epistemic uncertainty quantification, thereby supporting efficient and trustworthy collaboration between clinicians and data-driven decision-support tools. In the following, we summarize relevant work that quantifies epistemic uncertainty in medical use cases. We then describe our methodology: the dataset and the PCa mortality prediction task, the distinction between aleatoric and epistemic uncertainty in ML-based prediction, an overview of epistemic uncertainty estimation methods, the specific models used in this work, our approach to measuring epistemic uncertainty, and details on model training. Next, we present model performance on the PCa mortality prediction task and examine whether the instance-wise epistemic uncertainty measures correlate with predictive performance. To further interpret differences between model-specific uncertainty estimates, we analyze the models and their epistemic uncertainty behavior on a simple toy dataset. Finally, we discuss the results in the context of clinical applicability, highlighting both strengths and limitations to inform future research.

## 2. Methods

### 2.1. Ethics statement

This study utilized de-identified data from the Prostate, Lung, Colorectal, and Ovarian (PLCO) Cancer Screening Trial, which was approved by the institutional review boards of the U.S. National Cancer Institute (NCI) and all participating screening centers. All participants provided written informed consent prior to enrollment. The use of PLCO data in this analysis complies with the data use agreement and ethical guidelines established by the National Cancer Institute. Access to data was granted under project number PLCO-1797. The statements contained herein are solely those of the authors and do not represent or imply concurrence or endorsement by NCI.

### 2.2. Data

**Fig 1:**
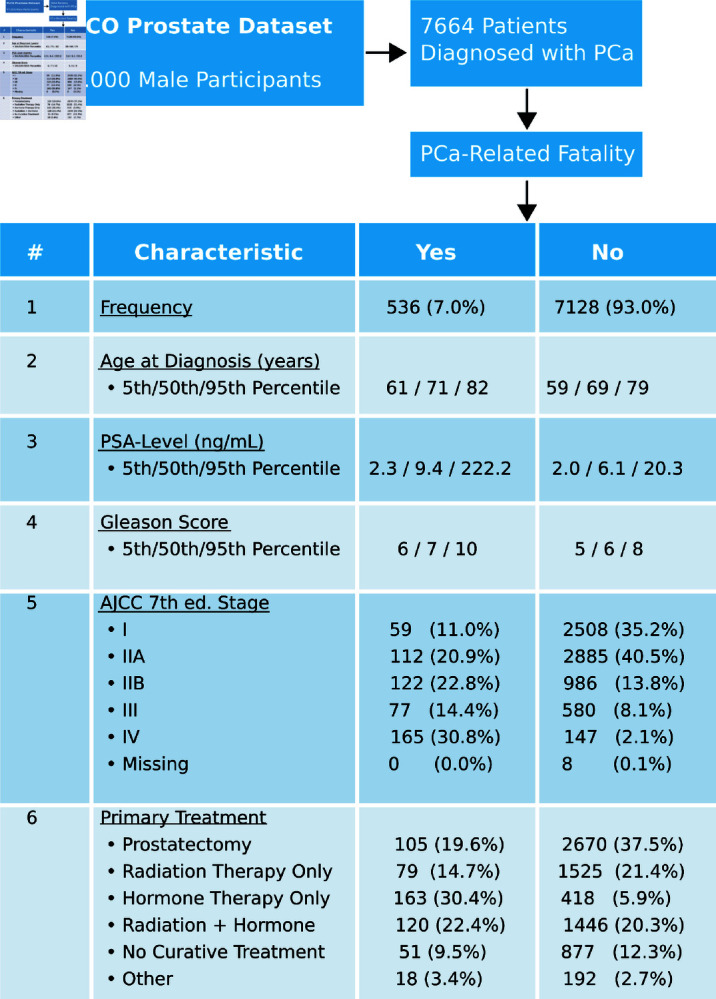
Selected statistics from the PLCO trial cohort considered for PCa mortality prediction.

The data used in this work was obtained from the Prostate, Lung, Colorectal, and Ovarian Cancer Screening Trial (PLCO) [56,57], a large randomized and controlled US-based study aimed to determine the effects of screening on cancer-related mortality and secondary endpoints. The PLCO prostate dataset comprises approximately 77.000 male participants, of whom 7.664 met our inclusion criteria (eligible for the baseline questionnaire, diagnosed with prostate cancer during the trial, and with complete follow-up). We excluded the PCa-diagnosed patients who were lost to follow-up or refused further contact. A short overview over the cohort is shown in Fig 1. From the considered cohort, 536 (7.0%) experienced a physician-reviewed, PCa–specific death within 13 years of initial diagnosis, 2474 (32.3%) died of other causes during the same period, and 4654 (60.7%) were still alive at 13 years post-diagnosis. We selected a 13-year horizon because it corresponds to the minimum follow-up time among all PCa survivors, eliminating any false negatives (no “still-alive” patient has had fewer than 13 years of observation). Moreover, 75% of prostate-cancer deaths within the trial occurred within these 13 years of initial diagnosis, so the cutoff captures the majority of events. Our binary prediction target is therefore “PCa–specific death within 13 years of diagnosis” (class 1) versus “no PCa–related death within 13 years” (class 0, including other-cause deaths and survivors). The study considers a variety of features such as lifestyle-related attributes, cancer history, comorbidities, extensive diagnostic information, as well as initial therapeutic procedures and follow-up characteristics. To select the most expressive features for prostate cancer mortality, the pair-wise $\phi_{k}$-correlation coefficient was considered [58]. $\phi_{k}$ captures non-linear dependencies and can be applied consistently to categorical, ordinal, and continuous variables. For subsequent PCa mortality prediction, only features with a correlation $\phi_{k}>0.03$ with PCa-related fatality and a corresponding p-value of *p*<0.05 were considered. Additionally, essentially redundant features with a strong pair-wise correlation (*ϕ_k_* > 0.9) to other chosen properties were omitted. This condensed the dataset to 34 suitable features including, among others, AJCC 7th Ed. staging, TNM-staging, PCa grade, PCa histopathologic type, Gleason score, PSA levels, primary treatment, cancer history, prior prostate-related medical events, various comorbidities such as stroke, heart attacks, osteoporosis, as well as lifestyle attributes, i.e. smoking habits or social participation. The extensive list of features used for model training can be found in the Appendix.

### 2.3. Epistemic uncertainty estimation and deep learning

In safety-critical applications we require *instance-wise* quantification of predictive uncertainty rather than population-level measures. In the context of machine learning, we distinguish two sources of predictive uncertainty [21,22,59]):

#### Aleatoric uncertainty.

Aleatoric, stochastic or data uncertainty arises from inherent randomness in the observations. Given a specific feature set, aleatoric uncertainty cannot be reduced by collecting more data points because it reflects fundamental variability in the underlying data-generating process, driven by unobserved latent factors. In ML-based classification, heteroscedastic aleatoric uncertainty is commonly captured by the softmax likelihood when optimizing for maximum likelihood [60]. In regression, aleatoric uncertainty can be captured either by explicitly predicting dispersion parameters of a chosen parametric output distribution [59] or by employing parameter-free techniques such as quantile regression [61].

#### Epistemic uncertainty.

Epistemic or knowledge uncertainty reflects limited knowledge about the true function. It arises due to insufficient data or inadequate assumptions of the model. Hüllermeier *et al*. [23] partition epistemic uncertainty into model uncertainty and approximation uncertainty. *Model uncertainty* arises when the hypothesis space of a chosen model cannot fully represent the true functional posterior. Classical machine-learning algorithms embed strong inductive biases—restrictive assumptions about the form of the learned function—that shrink this hypothesis space [23,62]. For example, decision trees and their ensembles (random forests, gradient boosting) yield piecewise-constant or piecewise-linear mappings; linear, logistic, and polynomial regressions impose specific algebraic relationships; naïve Bayes assumes feature-wise conditional independence; and nearest-neighbor methods, support vector machines, and Gaussian processes rely on locality or kernel-defined similarity measures on the input space that degrade in high-dimensional settings. These embedded biases systematically exclude large regions of the true posterior, causing model uncertainty to be masked and leading to underestimation of epistemic uncertainty. To achieve faithful epistemic uncertainty quantification, one must therefore minimize model uncertainty by employing more expressive hypothesis classes. Consequently, deep neural networks are the model of choice because, when sufficiently wide and deep, they can approximate any continuous function to arbitrary accuracy, yielding a highly expressive hypothesis space [23]. This capacity makes it likely that the relevant functions of the true posterior reside in or are well-approximated within the network’s hypothesis class, effectively minimizing model uncertainty. Employing such universal function approximators is therefore a prerequisite for trustworthy epistemic uncertainty estimation. To that end, Kendall and Gal [59] highlighted that epistemic uncertainty had been hard to capture in fields like computer vision precisely because traditional models lacked flexibility, but Bayesian deep learning now makes it feasible. With sufficiently expressive deep neural networks serving as universal function approximators, model uncertainty is typically considered negligible and may be safely ignored [23]. *Approximation uncertainty*, the second component of epistemic uncertainty, quantifies our ignorance about which function—among many that fit the observed data—is the “true” one, given only a finite dataset. Approximation uncertainty arises from limited data and imperfect inference within an otherwise sufficiently expressive model. It is greatest in regions of the input space poorly covered by training data and, in principle, diminishes as more representative data are collected.

### 2.4. Methods for epistemic uncertainty estimation

Multiple families of methods have been proposed to estimate epistemic uncertainty or to flag out-of-distribution (OOD) and anomalous inputs, but they vary widely in their underlying principles and reliability.

#### Heuristic, distance, and density-based approaches.

Classical density estimators—such as Gaussian mixture models or kernel density estimates—and deep generative models—like variational autoencoders [63] and normalizing flows [64]—attempt to learn the training-data distribution and use the resulting likelihood as a proxy for confidence. Distance-based schemes (e.g., deep k-nearest neighbors [65] or Mahalanobis-distance detectors [66]) compare new inputs to stored representations from the training set. Manifold-based techniques—including one-class classifiers [67], open-set recognition [68], and topological methods such as Morse networks [69]—learn a latent support for the data and test whether new samples lie within it. However, with the exception of probabilistic generative models (such as VAEs and normalizing flows), all these approaches rely on a deterministic mapping from each input to a single confidence or anomaly score, providing only point estimates of uncertainty, which can still be overconfident on OOD inputs. Indeed, even deep generative models have been shown to assign high likelihoods to unseen data points, failing to reliably separate in-distribution from OOD samples [43].

#### Approximate Bayesian inference.

The most principled approach to epistemic uncertainty quantification is to approximate the functional posterior distribution and to derive uncertainty from the dispersion of the posterior predictive. Two partially overlapping classes of methods achieve this:

- **Particle-based methods** generate multiple posterior samples. Markov chain Monte Carlo (MCMC) [70–72] is theoretically exact but often intractable for deep models. More scalable alternatives include independent deep ensembles [73,74] and jointly trained approaches like Monte Carlo dropout [75], batch ensembles [76], and Stein variational gradient descent (SVGD) [77–79]. In principle, particle-based methods perform a Bayesian model average by sampling from multiple posterior modes. In practice, however, scalable ensembles often collapse, with members converging to very similar solutions. Independently trained ensembles lack any explicit mechanism to encourage exploration of distinct functions. Consequently, any residual diversity stems only from random initialization, stochastic optimization, data subsampling, or variations in model architecture. Recent work [80] reveals that, because of their extreme flexibility, high-capacity neural networks often converge to very similar solutions, making it difficult to preserve ensemble diversity and avoid collapse. This finding highlights that generating genuinely diverse predictions in deep ensembles is inherently challenging. Even sophisticated, jointly trained particle methods such as SVGD have been shown to collapse toward the same posterior modes when their repulsive forces are applied in weight space [81,82].
- **Variational inference [83] and Laplace approximations [84]** both introduce a tractable family of weight distributions to approximate the true posterior. Variational methods optimize an evidence lower bound (ELBO) over a chosen distributional family, whereas the Laplace approximation fits a local Gaussian by performing a second-order Taylor expansion of the log posterior around its MAP estimate. Bayes by Backprop [85] is a prominent variational technique that uses backpropagation to learn a weight posterior in Bayesian neural networks. For scalability, often mean-field priors (e.g. factorized Gaussians) are adopted, but they have been shown to underestimate posterior variance between well-separated data regions [40]. A further challenge pronounced with mean-field priors is the ELBO’s inherent trade-off between likelihood fit and KL regularization: if weighted too heavily toward the prior, the model underfits; if too weak, the posterior becomes overly confident and underestimates epistemic uncertainty [41]. To remain tractable, also Laplace approximations adopt restrictive priors—commonly factorized Gaussian distributions over weights—which limit their ability to capture complex posterior structure [84]. Because the Laplace posterior is centered on a single mode, it cannot represent multiple, well-separated modes and thus may severely underestimate the volume of plausible hypotheses [42]. These shortcomings mirror those of mean-field variational inference: both approaches over-constrain the posterior, leading to overconfident predictions and underestimated epistemic uncertainty. These findings indicate that, despite their theoretical ability to approximate the posterior, particle-based and variational methods—when confined to weight-space and lacking explicit functional priors—often fail to explore the full range of plausible functions, since many distinct weight configurations can produce near-identical mappings.

When traditional weight-space methods fall short, recent research in Bayesian deep learning has shifted to approximations with explicit priors over functions, encouraging a richer diversity of plausible mappings. By using functional priors (VI) or particle-interactions in function space, they can enforce properties like distance-aware variance. For instance, functional Bayesian neural networks [86,87] augment standard weight priors with an auxiliary loss that aligns the induced function distribution to a target Gaussian-process prior. Deep Gaussian processes [88,89] stack GP layers to capture complex, hierarchical representations under a fully Bayesian treatment. Spectral-normalized neural Gaussian processes (SNGP) [90] marry a GP output layer with spectral normalization on hidden weights, preserving neural-network scalability while endowing the model with GP-like uncertainty guarantees. Beyond these, recent extensions apply function-space priors to Laplace approximations [91] or introduce repulsive interactions in function space for Stein Variational Gradient Descent [82], ensuring that ensemble members explore genuinely distinct functions. Collectively, these functional-prior methods deliver more faithful epistemic uncertainty estimates by directly regularizing the space of predictive functions.

In this work, we adopt approximate Bayesian deep learning as a principled framework for instance-wise epistemic uncertainty estimation. We evaluate two weight-space prior approaches—neural network ensembles (ENN) as a particle-based method and Bayesian neural networks (BNN) with factorized Gaussian weight priors as VI-based technique. We compare those implicit functional prior methods to spectral-normalized neural Gaussian processes (SNGP), which impose explicit, distance-aware priors directly in function space.

### 2.5. Bayesian mean reversion and uncertainty

Let $𝒟=\{(x_{i},y_{i}){\}}_{i=1}^{N}$ be i.i.d. (independent and identically distributed) training data, let x_*_ denote a test input and $f_{*}=f(x_{*})$ a corresponding model output. For predictive models whose prior encodes local correlation and distant independence, e.g., Gaussian processes with distance-decaying kernels, the posterior predictive at x_*_ will revert to the prior predictive whenever observations carry no information about the target variable within the kernel’s effective support, i.e. $p(f_{*}|𝒟)\to p(f_{*})$. Consequently, the posterior predictive mean satisfies $𝔼[f_{*}|𝒟]⟶𝔼[f_{*}]$.

To demonstrate, consider a model whose implicit prior over functions is a Gaussian process f(·)~GP(m(·),k(·,·)) with mean m(·) and a distance-decaying kernel k(·,·) (e.g. RBF or Matérn). For any subset ${𝒟}^{\prime }\subseteq 𝒟$ define $X^{\prime }=(x_{i}{)}_{(x_{i},y_{i})\in {𝒟}^{\prime }}$, $Y^{\prime }=(y_{i}{)}_{(x_{i},y_{i})\in {𝒟}^{\prime }}$, and $f_{X^{\prime }}=(f(x_{i}){)}_{(x_{i},y_{i})\in {𝒟}^{\prime }}$. Now consider a subset of the training data local to x_*_, $D_{\text{loc}}=\{(x_{i},y_{i})\in 𝒟:||x_{*}\;-\;x_{i}||\leq r\}$, and its complement $D_{\text{out}}=D\;⧵\;D_{\text{loc}}$, with the radius *r* chosen so that $k(x_{*},x_{i})>0\;\forall \;x_{i}\in D_{\text{loc}}$ and $k(x_{*},x_{i})\to 0\;\forall \;x_{i}\in D_{\text{out}}$. As a consequence of i.i.d., we may factorize the likelihood and the latent posterior becomes

$$p(f_{*},f_{X}|x_{*},D)=\propto p(f_{*},f_{X})p(Y_{\text{loc}}|f_{X_{\text{loc}}})p(Y_{\text{out}}|f_{X_{\text{out}}})\;==p(f_{*},f_{X})\prod_{(x_{i},y_{i})\in D_{\text{loc}}}p(y_{i}|f_{i})\prod_{(x_{i},y_{i})\in D_{\text{out}}}p(y_{i}|f_{i}).$$ (1)

In Eq (1), $p(Y_{\text{loc}}|f_{X_{\text{loc}}})$ and $p(Y_{\text{out}}|f_{X_{\text{out}}})$ denote the likelihood of the local and the outer training data subset, respectively. Now consider the following two extreme uncertainty regimes:

- **High epistemic uncertainty:** If no training data lies within radius *r* of x_*_, then ${𝒟}_{\text{loc}}=\emptyset$ and the local likelihood is constant with $p(Y_{\text{loc}}|f_{X_{\text{loc}}})=1$
- **High aleatoric uncertainty:** If local observations around x_*_ are fully noisy / ambiguous, the outputs *y*_*i*_ become statistically independent of *f*_*i*_. Equivalently, $y_{i}⟂\;\;\;⟂f_{i}$ or $p(y_{i}|f_{i})=p(y_{i})\propto 1\;\forall \;(x_{i},y_{i})\in {𝒟}_{\text{loc}}$

In both uncertainty extremes, the local likelihood terms collapse to constants. The joint latent posterior then simplifies to

$$p(f_{*},f_{X}|x_{*},𝒟)=p(f_{*},f_{X_{\text{out}}}|x_{*},{𝒟}_{\text{out}})\propto p(Y_{\text{out}}|f_{X_{\text{out}}})p(f_{*},f_{X}).$$ (2)

Next, let us demonstrate that the likelihood contributions from ${𝒟}_{\text{out}}$ also do not affect the posterior predictive distribution at x_*_. Under our kernel assumptions,

$$Cov(f_{*},f_{X_{\text{out}}})=0\;⟺\;f_{*}⟂\;\;\;⟂f_{i}\;\forall \;(x_{i},y_{i})\in {𝒟}_{\text{out}}$$ (3)

so the joint prior factorizes like

$$(\begin{array}{c} f_{*}\\ f_{X_{\text{out}}} \end{array})\sim 𝒩\left[(\begin{array}{c} m(x_{*})\\ m(X_{\text{out}}) \end{array}),(\begin{array}{cc} k(x_{*},x_{*}) & 0\\ 0 & 𝐊(X_{\text{out}},X_{\text{out}}) \end{array})\right]$$ (4)

Hence, the posterior predictive becomes

$$p(f_{*}|x_{*},𝒟)=p(f_{*}|x_{*})\sim 𝒩(m(x_{*}),k(x_{*},x_{*})).$$ (5)

Thus, in regimes of high epistemic or aleatoric uncertainty, the predictive distribution reverts to the prior predictive. These considerations are valid for the deep learning models considered in this work (BNN, ENN, and SNGP) and many other common Bayesian approaches to neural networks including MC dropout, Laplace approximations, and DeepGPs, as they can be understood to explicitly define or at least theoretically (under certain assumptions) implicitly approximate a Gaussian process prior over the model function [75,84,92–94]. However, the degree to which approximate Bayesian methods realize their ideal Gaussian-process behavior in practice depends critically on the quality of the approximation, the specifics of the optimization dynamics, and the chosen hyperparameter settings [40,74,85,95,96].

### 2.6. Sample-wise uncertainty measures in logit space

We consider measures of uncertainty directly in logit space, i.e. on the model outputs before applying any transformation to probabilities. By operating with logits, we aim to decouple uncertainty from the average prediction and prior class-frequency. We deliberately avoid information theoretic measures in probability space like Shannon entropy or mutual information, which peak at *p* = 0.5 and quantify the uncertainty of the outcome rather than the uncertainty in the prediction itself. Although the uncertainty measures considered are not new, we want to demonstrate how they follow directly from Bayesian principles via Bayes’ theorem:

$$p(y_{⋆}|x_{⋆})=\frac{p(x_{⋆}|y_{⋆})}{p(x_{⋆})}p(y_{⋆}).$$ (6)

In binary classification (y∈{0,1}), the Bayes factor (ratio of the likelihoods of the two possible outcomes) is

$$K=\frac{p(x_{⋆}|1)}{p(x_{⋆}|0)}=\frac{p(1|x_{⋆})}{p(0|x_{⋆})}\frac{p(0)}{p(1)}=e^{\log\frac{p(1|x_{⋆})}{p(0|x_{⋆})}-\log\frac{p(1)}{p(0)}}\equiv e^{\ell_{1|x_{⋆}}-\ell_{1}},$$ (7)

where $\ell_{1}$ represents the prior log-odds and $\ell_{1|x_{⋆}}$ the posterior log-odds. For a model with a single output, $\ell_{1|x_{⋆}}=f_{⋆}$; for a model with binary output, $\ell_{1|x_{⋆}}=f_{⋆,1}\;-\;f_{⋆,0}$, where *f* denotes the model function. Under i.i.d. and 1≪N, $\ell_{1}$ is estimated from training-set class frequencies with negligible uncertainty. Let $\{f_{⋆}^{\left(i\right)}{\}}_{i=1}^{M}$ with $f_{⋆}^{\left(i\right)}\sim p(f_{⋆}|𝒟,ℳ)\approx p(f_{⋆}|𝒟)$ be a collection of predictive functions drawn from the approximate posterior of model ℳ-e.g., an ensemble of neural networks (ENN) or sampled functions from a Bayesian neural network (BNN). We define the *Evidential Strength* (ES), which quantifies the strength of evidence in favor of one hypothesis over the other, as

$$ES\equiv \left|{𝔼}_{f_{⋆}\sim p(f_{⋆}|𝒟,ℳ)}[\log K(f_{⋆})]\right|=|{𝔼}_{f_{⋆}\sim p(f_{⋆}|𝒟,ℳ)}[\ell_{1|x}(f_{⋆})]\;-\;\ell_{1}|.$$ (8)

ES treats evidence for either hypothesis symmetrically and measures how far the Bayes factor deviates from neutrality (*K* = 1). Given mean reversion (see Sect 2.5), ES serves as a measure of total uncertainty (aleatoric + epistemic). For increased interpretability and visualization purposes, we apply a strictly monotone transformation into [0,1], yielding

$$u_{\text{tot}}\equiv e^{-kES},$$ (9)

where *k* is some pre-defined decay rate. The measure $u_{\text{tot}}$ is now bounded between 1 (maximum uncertainty, when the evidence equally supports both hypotheses or there is no evidence at all; *K* = 1) and 0 (minimum uncertainty, when there is overwhelming evidence for one hypothesis, K→0 or K→∞).

For measuring epistemic uncertainty we consider predictive dispersion, i.e. the overall spread or width of the predictive posterior distribution. This functional dispersion captures local sparsity of the evidential support for a given prediction and reveals the range of plausible model outputs consistent with the observed data, reflecting how the model’s knowledge varies across inputs. We utilize the variance of the posterior log-odds as follows:

$$u_{epi}\equiv 1-e^{-\;k\;{Var}_{f_{⋆}\sim p(f_{⋆}|𝒟,ℳ)}[\log K(f_{⋆})]}=1-e^{-\;k\;{Var}_{f_{⋆}\sim p(f_{⋆}|𝒟,ℳ)}[\ell_{1|x}(f_{⋆})]}.$$ (10)

This formulation, again employing an exponential decay transformation for interpretability, yields a maximum epistemic uncertainty of 1 for an infinitely wide functional posterior and a minimum of 0 for a delta-distributed posterior. Note, since SNGP provides a closed-form Gaussian posterior over the latent function, $p(f_{⋆}|𝒟)\sim 𝒩\left[f_{⋆};m_{𝒟}(x_{⋆}),k_{𝒟}(x_{⋆},x_{⋆})\right]$, we may avoid posterior sampling and use the latent mean and variance to compute our uncertainty measures.

### 2.7. Model training

Models (BNN, ENN, and SNGP) were optimized for the binary classification task of predicting mortality associated with prostate cancer following primary treatment. All models were trained in a supervised manner using the Adam optimizer [97]. Missing entries in the dataset were addressed by one-hot encoding of categorical variables and an auxiliary missing value flag for continuous variables. Cross validation on the dataset was carried out using stratified sampling, creating six-fold 4:1:1 train, validation, and test subsets with conserved frequency of the target variable. Early stopping [98] was implemented, evaluating the loss function on the hold-out validation set. All models consist of multiple fully-connected layers with point-weights (ENN, SNGP) or radially distributed weights (BNN) [99]. SNGP augments its residual layers with spectral normalization and implements the Gaussian-process output head using a random-Fourier-feature approximation. While for ENN and SNGP cross entropy loss was used as training objective, BNN was trained to maximize the ELBO (see appendix). To ensure sufficient statistical power, for ENN we employed 500 neural networks and 4096 random Fourier features for SNGP. All ensemble members shared the same architecture, but were differentiated by unique parameter initializations generated using the Kaiming uniform distribution [100]. An extensive grid search was conducted on model architecture, parameter initialization schemes and optimization hyperparameters.

## 3. Results

**Fig 2:**
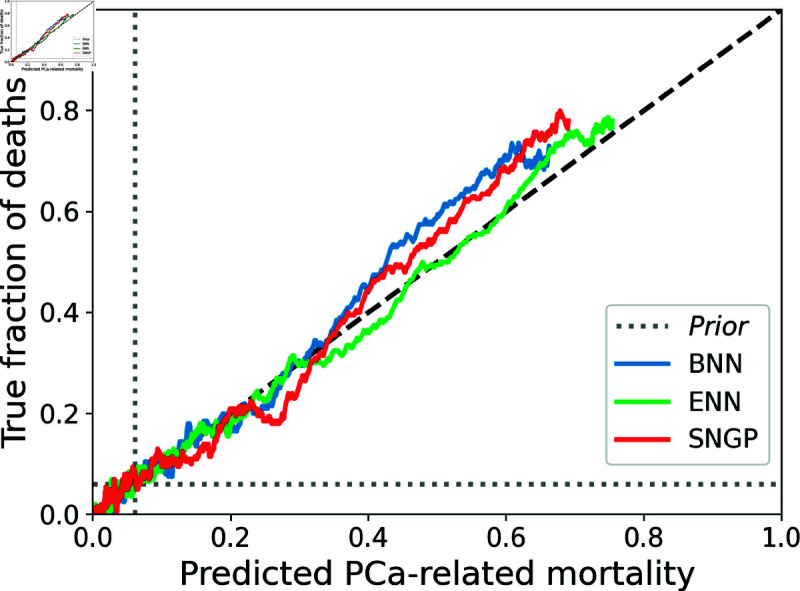
Calibration curves of the considered approximate Bayesian deep learning models in cross validation on the selected PLCO cohort.

In this study, we evaluated how well different approximate Bayesian deep learning methods quantify instance-wise epistemic uncertainty in a clinical prognosis setting. The quality of instance-wise uncertainty estimates produced by our models (BNN, ENN, and SNGP) was evaluated on the PLCO PCa mortality prediction task using six-fold cross-validation (details in Sect 2.7). As shown in Table 1, all three approaches achieved virtually identical discriminative performance measured by area under receiver operating characteristic curve (AUROC) and area under the precision-recall curve (AUPRC) and equivalent calibration measured by expected calibration error (ECE) and with calibration curves overlapping closely in Fig 2. Thus, at the population level, each model yields well-calibrated, non-overconfident risk estimates.

**Table 1:** PCa mortality stratification performance and calibration of the considered approximate Bayesian deep learning models in cross validation on the selected PLCO cohort measured in area under the receiver operating characteristic curve (AUROC, higher is better), area under the precision-recall curve (AUPRC, higher is better), and expected calibration error (ECE, lower is better).

Metric	BNN	ENN	SNGP
AUROC (μ±σ)	0.863 ± 0.009	0.857 ± 0.009	0.863 ± 0.009
AUPRC (μ±σ)	0.420 ± 0.029	0.423 ± 0.026	0.420 ± 0.029
ECE (μ±σ)	0.011 ± 0.002	0.011 ± 0.003	0.011 ± 0.003

**Fig 3:**
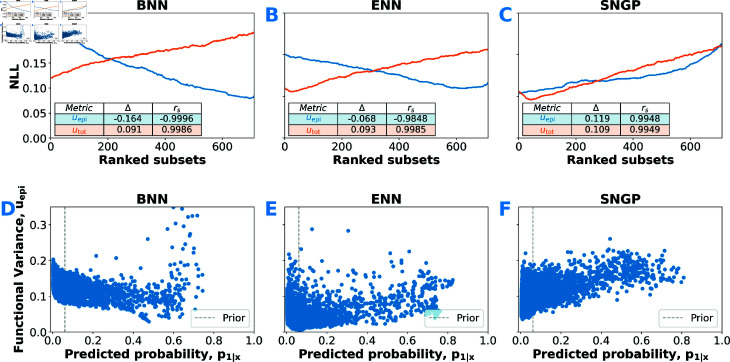
Stratification of predictive performance by uncertainty measures. *Top:* Negative log-likelihood (NLL, lower is better) computed on cross-validated PLCO-cohort subsets sorted by uncertainty rank (higher rank = higher uncertainty) for each approximate Bayesian deep learning model; *blue curves* denote epistemic uncertainty (Eq (10)), *orange curves* denote total uncertainty (Eq (9)). The inset tables report the difference in total NLL between the lowest- and highest-uncertainty subsets, along with Spearman’s rank correlation coefficient. *Bottom:* Epistemic uncertainty (Eq (10)) plotted against predicted PCa mortality (probability of PCa-related death).

Next, we asked whether this global calibration carries over to per-sample epistemic uncertainty. Fig 3(A)–3(C) report stratified negative log-likelihood (NLL) computed on sliding-window subsets (window size = one-third of the data, stride = 1), ranked by our total-uncertainty measure ($u_{\text{tot}}$, Eq (9)). For all models, higher $u_{\text{tot}}$ reliably identifies subsets with worse NLL with Spearman-ρ≈1), confirming that the total uncertainty measure effectively stratifies predictive performance. However, when we try to isolate epistemic uncertainty ($u_{\text{epi}}$, Eq (10)), only SNGP’s estimates correlate positively with NLL, while BNN and ENN show the opposite trend, with predictive accuracy actually improving for samples deemed “more epistemically uncertain". To understand this counter-intuitive behavior, we plotted $u_{\text{epi}}$ against predicted probability (Fig 3(D)–3(F)). Both BNN and ENN frequently assign high epistemic uncertainties to extreme probabilities (near 0 or 1) and lower uncertainty in the mid-range—a direct contradiction of the notion that confident predictions (away from the prior frequency) should exhibit low epistemic uncertainty. SNGP, by contrast, shows no such artifact and its $u_{\text{epi}}$ estimates well-stratify predictive performance, suggesting, out of the three models considered, it alone captures genuine uncertainty related to regions poorly supported by training data.

**Fig 4:**
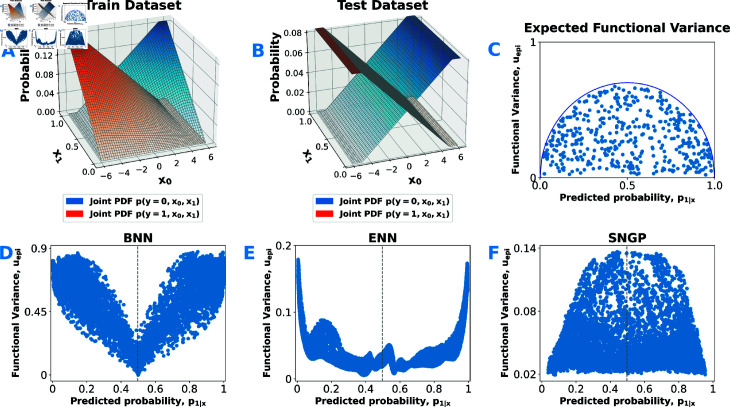
Epistemic uncertainty measures (Eq (10)) on toy data. *(A)* Training data: class probability varies linearly along *x*_0_, and data density changes linearly along *x*_1_. *(B)* Test data: class probability varies linearly along *x*_0_, with uniform data density. *(C)* Expected schematic scatter plot of epistemic uncertainty versus predicted probability, illustrating Bayesian mean reversion (Sect 2.5). *(D–F)* Scatter plots of epistemic uncertainty versus predicted probability for each approximate Bayesian deep learning model considered.

Because co-related data sparsity and class-overlap patterns in the PLCO data might confound these observations, we constructed a controlled two-dimensional binary classification toy dataset whose true class frequency varies linearly along one input axis, while data point density varies along the other (Fig 4(A)). In this dataset, we deliberately made the target frequency statistically independent of data density, thereby fully disentangling epistemic uncertainty from aleatoric uncertainty in the ground truth. We trained each model on this data distribution and then evaluated on a similar distribution, but with uniform data density across the entire input domain (Fig 4(B)). We expect the ideal Bayesian predictor to “mean-revert” (see Sect 2.5), i.e. drive uncertain predictions toward the prior 0.5—producing the schematic in Fig 4(C). In practice (Fig 4(D)–4(F)), only SNGP exhibits clear mean reversion, while BNN and ENN again invert this pattern, peaking at extreme predicted probabilities.

**Fig 5:**
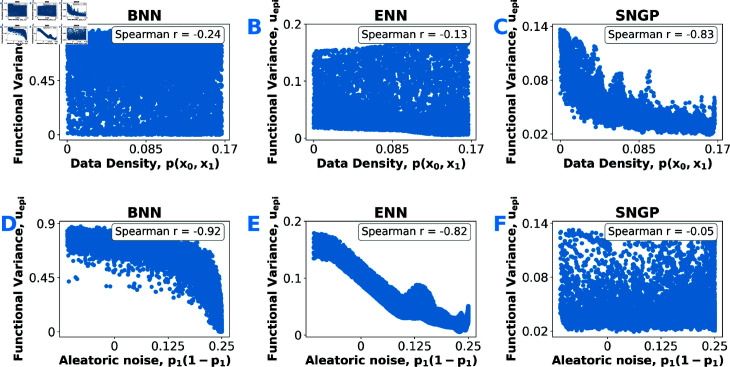
Epistemic uncertainty measures (Eq (10)) versus data characteristics on toy data. *(A–C)* Epistemic uncertainty plotted against data density $p(x_{0},x_{1})$ for each approximate Bayesian deep learning model considered. *(D–F)* Epistemic uncertainty versus label noise $p_{1}(1-p_{1})$ for each approximate Bayesian deep learning model considered. Each panel includes the Spearman’s rank correlation coefficient.

Finally, we evaluated how each model’s epistemic uncertainty aligns with data density versus aleatoric noise. Ideally, epistemic uncertainty would rise where data are sparse and remain unaffected by aleatoric variability. In Fig 5(A)–5(C), SNGP’s $u_{\text{epi}}$ correlates strongly with local data density (higher uncertainty where data are sparse), while BNN and ENN show much weaker density dependence. Conversely, Fig 5(D)–5(F) reveals that BNN and ENN’s $u_{\text{epi}}$ correlates strongly with label noise (aleatoric uncertainty), while SNGP remains essentially insensitive. In other words, BNN and ENN conflate aleatoric and epistemic uncertainty—explaining their inverted stratification of predictive performance in Fig 3(A) and 3(B): ranking by their epistemic uncertainty estimates ends up stratifying samples by data noise. Only SNGP’s strong alignment with data density on the toy data (and its independence from aleatoric noise) indicates that its epistemic uncertainty estimates truly stratifies predictive performance based on data support. In other words, SNGP genuinely measures how much evidence it has seen for each sample.

## 4. Discussion

Across the population-level metrics, all three models exhibit virtually indistinguishable discriminative performance and near-perfect probability calibration on the PLCO-based PCa-mortality prediction task (Table 1 and Fig 2). Because the cross-entropy loss employed during training is a strictly proper scoring rule, optimizing it (in expectation) guarantees calibration with respect to aleatoric noise [101]. Consequently, any remaining miscalibration must essentially originate from epistemic uncertainty. Indeed, prior work has shown that miscalibration in deep networks is driven by model-based factors rather than data noise [16], and that calibration degrades under distributional shift precisely when epistemic uncertainty grows [102]. One might therefore infer that our dataset harbors little epistemic risk, given the excellent population-level calibration. However, when we stratify predictive performance by per-sample epistemic uncertainty estimates from the SNGP model, we uncover cases with substantial epistemic uncertainty that remain hidden in aggregate calibration. This demonstrates that strong overall calibration does not guarantee low epistemic risk on individual predictions, underscoring the importance of high-fidelity, instance-wise uncertainty estimates.

While SNGP’s accuracy improved when epistemically uncertain predictions were excluded, both BNN and ENN failed to show this benefit—in fact, their performance worsened—prompting us to investigate their uncertainty estimates more closely. We discovered a pronounced bias in both models’ epistemic uncertainty estimates. The functional dispersion measure $u_{\text{epi}}$ showed substantially greater variability for inputs with extreme predicted probabilities ($p_{1|x}\to 0$ or 1) than for those with more moderate probabilities, across both the PLCO task and our toy dataset. In the controlled toy experiments that eliminate dataset-specific artifacts, this bias not only persisted but became even more pronounced. We hypothesize that, in both BNN and ENN, functional variance estimates become entangled with aleatoric uncertainty because the sigmoid/softmax link functions combined with the cross-entropy loss determine the local curvature of the loss surface. For example, under a Laplace-style (second-order) approximation and neglecting regularization effects, the logit variance scales as

$$Var[l_{1|x}]\propto \frac{1}{p_{1|x}(1-p_{1|x})}.$$ (11)

Logits at the probability extremes display artificially inflated variance, because perturbations in these regions produce minimal changes in loss. Conversely, for logits close to the prior (e.g., $p_{1|x}\to 0.5$ in our toy example), the loss function is highly sensitive to change. In other words, the nonlinearity that shapes the loss surface forces logit-space variance to mirror the data-intrinsic noise, entangling epistemic estimates with aleatoric uncertainty. Switching to probability-space dispersion (using information-theoretic measures) does not remedy the problem, because probability-space gradients likewise vanish at the tails. Thus, for both BNNs and ENNs, neither logit- nor probability-space dispersion provides an unbiased estimate of epistemic uncertainty. Instead, these measures strongly correlate with aleatoric noise—echoing prior findings that many so-called epistemic scores are confounded by data-inherent variability [103,104]. While both BNN and ENN epistemic estimates are entangled with aleatoric uncertainty, they correlate only weakly with local data density, indicating a fundamental misestimation of functional ambiguity. In ENNs, independently trained members fail to diversify sufficiently on out-of-distribution inputs, preventing adequate exploration of the functional posterior [77]. Lindenmeyer *et al*. [105] demonstrated that ReLU-activated networks effectively act as piecewise-linear interpolators, relying on simple linear interpolation between neighboring data points. Factorized variational approaches—including our BNN and Laplace variants—exhibit the same limitation when interpolating across data gaps [106]. Moreover, as non-cooperative collections of predictors, ENNs lack any mechanism (such as Bayesian mean reversion) to pull uncertain predictions back toward prior expectations. Even when member functions diverge in sparse regions, the ensemble mean remains governed by boundary trends. Similarly, although BNNs impose weight-space priors, these do not enforce functional extrapolation behaviors towards prior beliefs [86]. Although these functional priors can be encoded at initialization in both BNNs and ENNs, gradient–based training offers no guarantee that they will be preserved through optimization. As a result, neither ENN nor BNN accurately approximates the true functional posterior—undermining not only $u_{\text{epi}}$, but also our total uncertainty measure $u_{\text{tot}}$. While $u_{\text{tot}}$ successfully stratifies performance (Fig 3(A) and 3(B)), it is essentially dominated by aleatoric variance only.

In SNGP, the logit-space variance $u_{\text{epi}}$ comes from the GP predictive variance—driven by a distance-aware kernel over feature representations—rather than loss curvature, so its functional-variance estimates depend solely on proximity to training data and remain independent of aleatoric noise. Indeed, we observed that SNGP’s functional-variance estimates align closely with local data density and remain decoupled from aleatoric noise. This behavior is expected, since the Gaussian process posterior variance depends only on the covariance structure and not on the learned mean function. By integrating deep representation learning with a Gaussian-process–style Bayesian approximation, SNGP explicitly encodes distance-aware priors and enforces mean reversion [90], which together promote exploration of the functional posterior. As a result, even though all models achieved similar population-level metrics, SNGP carries substantially lower epistemic risk for clinical decision support than either BNN or ENN. These observations concur with prior work showing that richer posterior approximations and explicit, distance-sensitive functional priors yield markedly better epistemic-uncertainty estimates than methods lacking guaranteed functional variability [54,55,90].

### Implications.

This study carries significant implications for data-driven CDS systems and collaborative clinical decision making. In safety-critical domains such as clinical decision support, automation must lighten routine burdens without relinquishing human oversight. Sample-specific epistemic uncertainty estimation is therefore essential: by transparently communicating a model’s knowledge boundaries on a per-case basis, we enable clinicians to recognize when to trust automated predictions and when to consult their own or additional expertise. Also in ensemble or modular AI systems (e.g., digital twins or expert-backboned language models), accurate, per-component epistemic confidence scores ensure that each module contributes only where it is most reliable. Approximate Bayesian methods with implicit priors (e.g., standard BNNs and ENNs) offer no guarantees that their epistemic uncertainty scores faithfully reflect evidential support. Methods with explicit, distance-aware functional priors (as for example in SNGP) furnish epistemic uncertainty estimates that depend solely on proximity to training data and promise increased exploration of the functional posterior. Without high-quality, per-sample epistemic uncertainty estimates, practitioners cannot reliably distinguish trustworthy recommendations from spurious ones—undermining trust, risking patient harm, and forcing unnecessary review of low-risk cases. Conversely, robust uncertainty quantification allows clinicians to act confidently on low-risk predictions while focusing their expertise on genuinely uncertain cases, thereby optimizing both safety and efficiency in personalized, data-driven medicine. However, existing regulatory frameworks and AI-specific guidelines do not adequately support this vision of trust-based collaboration. Medical-device regulations and AI standards mandate only population-level calibration, discrimination, and clinical validity [18–20], metrics that—as our results show—can be misleading and fail to guarantee reliable individual predictions. To bridge this gap, regulatory benchmarks should be expanded to include rigorous evaluation of sample-wise uncertainty quantification, ensuring that clinical decision-support systems meet the demands of real-world, safety-critical deployment.

A clear separation of aleatoric and epistemic uncertainty is critical because they carry fundamentally different implications for decision making. Aleatoric uncertainty reflects inherent data noise or conflicting evidence—situations where additional review rarely changes the outcome—whereas epistemic uncertainty signals a genuine lack of knowledge about a particular case, making human judgment indispensable. In practice, clinical decision-support CDS tools should therefore present decoupled epistemic-uncertainty scores alongside their prognosis probabilities (for example, via numeric scores or simple “traffic-light” thresholds). Given models that include Bayesian mean reversion (Sect 2.5), such as SNGP, these two outputs alone suffice to convey both the prediction and the degree of evidential support behind it. Applied to PCa mortality prediction this might look like:

- *High confidence* ($p_{1|x}\to 0$ or 1, low $u_{\text{epi}}$): the model’s prediction is well supported; clinicians and patients can use it directly to guide decisions on PCa treatment aggressiveness.
- *Epistemic uncertainty* ($p_{1|x}\approx p_{1}$, high $u_{\text{epi}}$): the patient lies outside the model’s experience; here, clinicians should rely on their expertise or seek additional data before deciding on PCa intervention.
- *Aleatoric ambiguity* ($p_{1|x}\approx p_{1}$, low $u_{\text{epi}}$): many similar cases are known, but outcomes are split—review adds little value and unfortunately no clear recommendation of PCa therapy emerges.

Of course, many real-world cases fall between these extremes, exhibiting mixed levels of aleatoric and epistemic uncertainty that demand nuanced interpretation. By focusing human review on the most epistemically uncertain cases while safely allowing more automation for high-confidence ones, we both improve efficiency and ensure that expert judgment is applied where it matters most.

### Limitations.

Several limitations warrant consideration. First, our experiments were confined to particular architectures, prior specifications, and training protocols for BNN, ENN, and SNGP. Although we conducted an extensive hyperparameter search, our results remain evidential rather than definitive, and we do not claim a conclusive ranking of approximate Bayesian methods. Second, in line with prior work, we observed that explicit, distance-aware methods (like SNGP) deliver superior epistemic-uncertainty fidelity compared to implicit-prior approaches (BNN, ENN), and that the difference between these two groups exceeds the variability seen within each group. Nevertheless, because we tested only a limited set of representative models, we cannot claim this finding as universally conclusive. Our goal was to illustrate, using a selection of well-known and promising techniques, the advantage of instance-wise uncertainty metrics over population-level measures and the benefits of guaranteed posterior properties for epistemic fidelity. We do not dismiss the potential of BNN or ENN to yield accurate uncertainty estimates, but we highlight the inherent challenge of enforcing functional priors in data-sparse regions without explicit mechanisms. Finally, our analysis was restricted to a single real-world prognostic task (PCa mortality) and a controlled synthetic dataset; future work should assess generalization to other domains and architectures.

## 5. Conclusions

In this work, we have demonstrated that, despite comparable population-level accuracy and calibration, common approximate Bayesian deep learning methods—neural network ensembles and factorized weight-prior Bayesian neural networks—fail to deliver reliable, per-case estimates of epistemic uncertainty. Their functional variance measures become systematically biased by loss curvature, conflating epistemic risk with aleatoric noise and overlooking regions where the model truly lacks evidence. By contrast, spectral-normalized neural Gaussian processes, which enforce explicit, distance-aware functional priors and mean reversion, recover uncertainty estimates that align tightly with data sparsity and remain decoupled from stochastic noise. These findings carry important implications for safety-critical clinical decision support. Accurate, instance-wise epistemic uncertainty quantification is essential for guiding human–AI collaboration—enabling clinicians to trust predictions when evidence is strong and to intervene when the model’s knowledge is limited. Current regulatory standards, which focus on aggregate metrics, do not guarantee such granular reliability. We therefore advocate incorporating sample-wise uncertainty benchmarks into AI-based medical device evaluation and prioritizing architectures with explicit functional priors for deployment in clinical settings. Looking forward, these results motivate further exploration of functional-prior methods—such as deep Gaussian processes, functional Bayesian neural networks, and other hybrid architectures—to ensure trustworthy uncertainty communication across a broad range of medical applications. By combining rich posterior approximations with transparent, per-case confidence scores, we can empower practitioners to harness the full potential of data-driven decision support while safeguarding patient safety.

## 6. Appendix

### 6.1. Feature set

See Table 2.

**Table 2:** Selected features from the PLCO prostate dataset used for PC mortality prediction.

RoyalBlue Feature name	Description
pros_stage_7e	Stage (AJCC 7th edition)
pros_stage_t	Prostate stage T component
pros_stage_m	Prostate stage M component
pros_stage_n	Prostate stage N component
pros_grade	Prostate cancer grade
pros_gleason	Gleason score
primary_trtp	Primary treatment
ph_first_cancer	First personal history of cancer
ph_pros_muq	MUQ analysis of prostate cancer
ph_any_muq	MUQ analysis of any cancer
reassympp	Symptomatic at initial clinical assessment
reassurvp	Reason for initial clinical assessment
reasothp	Existance of other reason for initial clinical assessment
pros_histtype	Prostate cancer histopathologic type
occupat	Occupation
rectal_history	Had a digital rectal exam during the past 3 years
intstatp_cat	Prostate cancer screen based on detected/interval status
urinatea	Age at which began waking up to urinate more than once at night
cig_years	Duration patient smoked cigarettes
bmi_curc	BMI at baseline (in lb/in2)
psa_history	Had blood test for prostate cancer in past 3 years
enlprosa	How old when told had enlarged prostate or BPH?
stroke_f	Did patient have a stroke
hearta_f	Had coronary heart disease or a heart attack
infpros_f	Ever had inflamed prostate?
arthrit_f	Ever had arthritis?
osteopor_f	Ever had osteoporosis?
vasect_f	Had a vasectomy?
pipe	Ever smoked a pipe for a year or longer?
marital	Marital status
race7	Race
surg_age	Age at first prostate surgery
surg_resection	Ever had transurethral resection of prostate?
infprosa	How old when told had inflamed prostate?
